# Decreased PGE_2_ Content Reduces MMP-1 Activity and Consequently Increases Collagen Density in Human Varicose Vein

**DOI:** 10.1371/journal.pone.0088021

**Published:** 2014-02-05

**Authors:** Ingrid Gomez, Chabha Benyahia, Liliane Louedec, Guy Leséche, Marie-Paule Jacob, Dan Longrois, Xavier Norel

**Affiliations:** 1 INSERM, U698, Paris, France; 2 University Paris Nord, UMR-S698, Paris, France; 3 AP-HP CHU X. Bichat, Department of Anesthesia and Intensive Care, University Paris Diderot, Sorbonne Paris*-*Cité, UMR-S698, Paris, France; 4 AP-HP CHU X. Bichat, Department of Vascular and Thoracic Surgery, University Paris Diderot, Sorbonne Paris-Cité, UMR-S698, Paris, France; University of Louisville, United States of America

## Abstract

Varicose veins are elongated and dilated saphenous veins. Despite the high prevalence of this disease, its pathogenesis remains unclear.

**Aims:**

In this study, we investigated the control of matrix metalloproteinases (MMPs) expression by prostaglandin (PG)E_2_ during the vascular wall remodeling of human varicose veins.

**Methods and Results:**

Varicose (small (SDv) and large diameter (LDv)) and healthy saphenous veins (SV) were obtained after surgery. Microsomal and cytosolic PGE-synthases (mPGES and cPGES) protein and mRNA responsible for PGE_2_ metabolism were analyzed in all veins. cPGES protein was absent while its mRNA was weakly expressed. mPGES-2 expression was similar in the different saphenous veins. mPGES-1 mRNA and protein were detected in healthy veins and a significant decrease was found in LDv. Additionally, 15-hydroxyprostaglandin dehydrogenase (15-PGDH), responsible for PGE_2_ degradation, was over-expressed in varicose veins. These variations in mPGES-1 and 15-PGDH density account for the decreased PGE_2_ level observed in varicose veins. Furthermore, a significant decrease in PGE_2_ receptor (EP4) levels was also found in SDv and LDv. Active MMP-1 and total MMP-2 concentrations were significantly decreased in varicose veins while the tissue inhibitors of metalloproteinases (TIMP -1 and -2), were significantly increased, probably explaining the increased collagen content found in LDv. Finally, the MMP/TIMP ratio is restored by exogenous PGE_2_ in varicose veins and reduced in presence of an EP4 receptor antagonist in healthy veins.

**Conclusions:**

In conclusion, PGE_2_ could be responsible for the vascular wall thickening in human varicose veins. This mechanism could be protective, strengthening the vascular wall in order to counteract venous stasis.

## Introduction

Varicose saphenous veins are characterized by venous backflow and blood stagnation. [1], [2], [3], [4] This pathology is part of the chronic venous disease of the legs that is categorized into several classes from C0 to C6, where the C2 stage corresponds to varicose veins which are frequently removed by surgery. [5] Despite the high incidence of this disease, its pathogenesis is still poorly understood although some hypotheses, such as a local hypertension or a genetic predisposition, have been suggested. [2], [3].

The metabolism and the effects of bioactive lipids like prostanoids (prostaglandins (PG) and thromboxane) has been rarely investigated in the context of varicose veins. Prostanoids are produced by most blood and vascular cell types. [6] PGE_2_ *via* selective activation of EP1-4 receptor subtypes is involved in the control of vascular tone, [7], [8] inflammation, [9], [10], [11], [12] pain [13] and vascular wall remodeling. [14] PGE_2_ is synthesized from arachidonic acid (AA) through the enzymatic activities of two cyclooxygenases (COX-1 and/or COX-2) and three PGE synthases (PGES). [15] Furthermore, PGE_2_ is degraded by 15-hydroxyprostaglandin dehydrogenase (15-PGDH), the only enzyme responsible for its catabolism. Among the three PGES that specifically catalyze the final step of PGE_2_ biosynthesis; two are constitutive: microsomal (mPGES-2) and cytosolic (cPGES). [12], [16] The third, mPGES-1, [11], [12], [16], [17], [18], is quantitatively the most important enzymatic activity for PGE_2_ production. mPGES-1 and COX-2 expression are generally co-induced by inflammatory cytokines such as IL-1β. [11], [17] However, in a recent publication, [19] we have shown the absence of COX-2 in the varicose veins.

As observed during aneurysm formation or in the pathogenesis of endometriosis, [14], [20], [21], [22] PGE_2_ modulates vascular wall remodeling mediated by the matrix metalloproteinases (MMPs). The renewal of extracellular matrix (ECM) by MMP [23] activity is dysregulated in many vascular diseases [24] such as acute coronary artery syndrome, atherosclerosis or aneurysm. [25], [26], [27], [28], [29] Some MMPs involved in these processes are the interstitial collagenase, MMP-1, that cleaves fibrillar collagens, which are subsequently degraded by the gelatinases, MMP-2 and MMP-9. [25], [28] There are few studies in human tissues which have demonstrated the role of PGE_2_ on the expression/activation of MMPs. [14], [20], [21], [22] For example, PGE_2_ activates several MMPs *via* EP2/EP4-receptor stimulation in human endometriotic epithelial and stromal cells. [14] On the other hand, MMP activities are also under control of endogenous tissue inhibitor of metalloproteinase (TIMP) and changes in MMP/TIMP ratio are probably involved in vascular wall remodeling and in varicose vein formation. [30], [31], [32], [33], [34].

The aim of this study was to investigate the role of PGE_2_ in the mechanism involved in the formation of varicose veins. We have investigated how PGE_2_ and the enzymes responsible for its metabolism could participate in venous wall remodeling *via* MMP, TIMP and collagen deposition. Specifically, this study was designed to analyse the pathology of varicose veins. For each patient with varicose veins (C2 stage) removed by surgery, we compared a dilated segment and a non-dilated segment. These two samples were compared with healthy saphenous veins (SV). An increase in diameter between segments of the healthy SV and the lesser dilated varicose vein can be observed. Therefore, our hypothesis is that the non-dilated varicose vein segment represents an intermediate stage of the disease. In this way, our study addresses the evolution of varicose pathology.

## Methods

### 1 Human Saphenous Veins (Healthy and Varicose)

Fifteen healthy SV from calf patients undergoing bypass surgery (10 male and 5 female, aged 71±4 years) were obtained. In most European countries, varicose veins are still frequently removed by conventional surgery. Varicose veins (n = 30, stage C2) from patients undergoing vein stripping (14 male and 16 female, aged 59±4 years) were obtained at Bichat Hospital (Paris, France) with patients’ verbal and written informed consent. The consent form, explaining all the procedure, was approved by the Bichat ethic committee. The investigation conforms to the principles outlined in the declaration of Helsinki as these tissues were anonymized. All research programs involving the use of human tissue were approved and supported by the National Institute for Health and Medical Research (INSERM) ethics committee and these tissues are considered as surgical waste in accordance with French ethical laws (L.1211-3–L.1211-9). Following harvesting, veins were rapidly placed in ice-cold saline. After surgical use, non-used healthy SV (∼3 mm) or varicose vein segments were immediately transported to the laboratory. All veins were processed within two hours after excision. They were cleaned of adipose tissue and blood. Varicose veins were separated into two different segments, of small diameter (SDv, ∼4 mm) and of large diameter (LDv, ∼8 mm). Saphenous veins obtained after bypass surgery are considered as healthy since patients were not under NSAIDs medication and veins were checked using sonographic ultrasound studies. All preparations were used with intact adventitial layers.

### 2 Saphenous Vein Preparation and Incubation

For Western blotting, fresh vein samples were ground in liquid nitrogen and homogenized in a RIPA solution as previously described. [19] After a 15 min centrifugation at 4500 g, the supernatant was stored at −20°C. Proteins were quantified using a bicinchoninic acid (BCA) assay kit (ThermoScientific, Rockford, IL, USA). For histological studies, fresh veins were fixed for 24 h in paraformaldehyde (3.7% in PBS), embedded in paraffin and sectioned at 5 µm. Sections were deparaffinized in toluene and hydrated at the beginning of the experiments. Before enzyme immunoassay (EIA) measurement of PGE_2_, MMPs or TIMPs, fresh vein samples were incubated at 37°C either for 24 h (70 mg wet weight tissue/mL solution) in RPMI solution (Gibco Invitrogen, Paisley, UK) with 5% CO_2_ or for 30 min in Tyrode’s solution.with 5% CO_2_ in O_2_. RPMI solution always contained antibiotics (penicillin, 1000 IU/mL; streptomycin, 100 µg/mL) and antimycotic (amphotericin, 0.25 µg/mL). The fresh venous preparations were incubated for 24 h in RMPI+antibiotics solution with or without pharmacological treatments (EP receptor agonist or antagonist). The fresh venous preparations were incubated for 30 min in Tyrode’solution with or without (arachidonic acid +/− glutathione). Supernatants were harvested and frozen until use.

### 3 Western Blot Analysis

Protein analysis was evaluated as previously described. [19] Fifty micrograms of venous proteins or Western ready control (for all proteins, from Cayman, Ann Arbour, MI, USA) were used and specific antibodies are presented in the Methods section of Supplementary data.

### 4 EIA

#### 4.1 Measurement of PGE_2_ levels

The concentration of PGE_2_ in the supernatant of healthy SV or varicose veins was determined using an enzyme immunoassay (EIA) kit (Cayman). Frozen supernatants were used and diluted with the assay buffer. The PGE_2_ concentration was determined according to the instructions provided with the kit. In addition, 30 min incubations in presence of arachidonic acid (1 µmol/L, Cayman) and with or without glutathione (2.5 mmol/L, Sigma) [11] were also performed (data not shown).

#### 4.2 Measurement of MMP and TIMP levels

MMP and TIMP protein levels were measured in the supernatant of vascular preparations using commercial kits (DuoSet, R&D systems, Minneapolis, MN, USA). Tissues were incubated in absence or presence of PGE_2_ (10 µmol/L, Cayman) or an EP4 receptor antagonist GW627368X [35] (1 µmol/L, Cayman). Results were expressed in pg or ng per mg of wet tissue. For MMP-1, measurements were made by using two different kits. The first kit recognized only the pro MMP-1 and the second kit recognize the total MMP-1 (pro-MMP-1 and active MMP-1 together). The quantity of active MMP-1 was calculated by the subtraction of pro-MMP-1 value to total MMP-1 value for each sample. MMP-2 was measured using kit which recognizes total MMP. TIMP-1 and TIMP-2 concentrations were measured using kits (one for TIMP-1 and another one for TIMP-2), which recognize the total human TIMP-1 or -2 (free and complexed with MMPs).

### 5 Histological Study

Masson’s trichrome staining was used for detection of several collagen and general morphology [36] of the healthy and varicose veins.

### 6 Data Analysis

Due to the specificity of the experiments, several normalization procedures were performed (Supplementary data). For specific protein content measured by Western blot, the level of protein expression was normalised by the α-actin content. Optical density (OD) for the different bands was measured by Scion Image®. For EIA measurements, normalisation was done by correcting for tissue wet weight. Collagen content were measured by calculating the optical density (pixel number) with Photoshop® and normalized by the total area of the vein sections.

Statistical analyses were performed using the program SigmaStat® (Systat Software, IL, USA). All data are presented as means ± sem derived from (n) patients. A one-way-ANOVA test (followed by the Tukey post-hoc test) or a paired t-test was used and a P-value <0.05 was considered as statistically significant. Linear regression and the Pearson test were used for correlation analysis (SigmaStat®).

## Results

### PGES and COX Content in Varicose and Healthy Saphenous Veins

**Figure 1:**
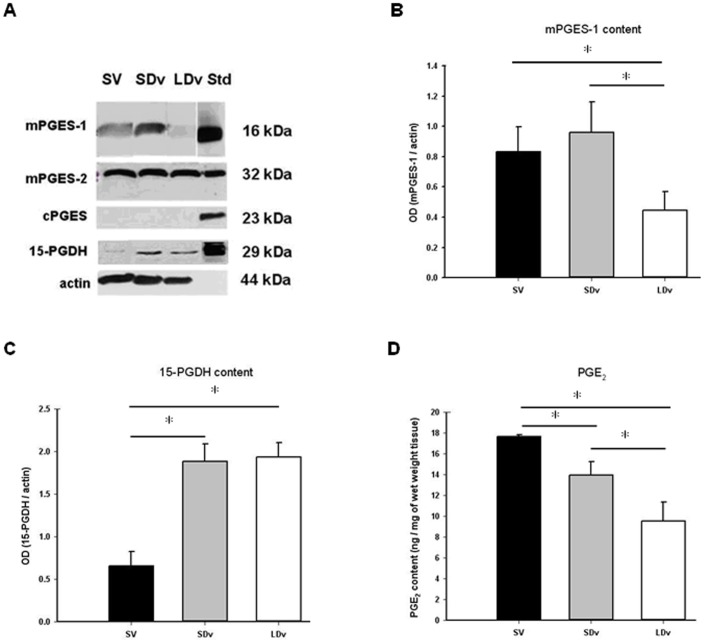
Dysregulation of PGE_2_ synthesis. Protein measurements, representative samples of Western blot (A) of microsomal and cytosolic prostaglandin E synthases (mPGES-1, mPGES-2 or cPGES), 15-hydroxyprostaglandin dehydrogenase (15-PGDH) in human small and large diameter varicosities (paired SDv and LDv, n = 6) and healthy saphenous veins (SV, n = 4). Standards (Std) are Western ready controls from Cayman. Histograms represent Western blot quantification of mPGES-1 (B) and 15-PGDH (C) corresponding bands. Optical density (OD, arbitrary units) was quantified by Scion Image® and the mean normalized by actin. PGE_2_ content (D) in paired SDv and LDv (n = 8) and healthy saphenous veins (SV, n = 4) was determined by EIA in supernatants after 24 h of incubation of the venous preparations in RPMI solution. Results are normalized by tissue wet weight. * P<0.05 as determined by one-way-ANOVA followed by the Tukey post-hoc test and by a paired t-test for varicose veins.

Expression of PGES isomers was measured using Western blot analysis and normalized to α-actin (Figures 1 A, B); for mPGES-1, a 16 kDa band was found in homogenates of healthy SV (n = 4) and varicose veins (n = 5) corresponding to the standard band. A significant decrease (about 50%) for mPGES-1 intensity was observed in the LDv samples as compared to the healthy SV. A 32 kDa band was found for mPGES-2 without modification of its intensity (n = 4–5) among the different venous preparations. cPGES was not detected in venous tissues, even using a greater protein load (100 µg) or another antibody for cPGES (n = 6–7). In order to confirm this result, Western blot analysis with protein extracts derived from human internal mammary artery was performed and a 23 kDa band corresponding to this isoform was detected (Figure S1). Furthermore, measurement of mPGES-1 transcript (Figure S2 and Table S1 in the File S1) confirmed the decreased expression (about 75%) of this enzyme in LDv (n = 5). In all venous preparations, mPGES-2 and cPGES mRNA were expressed (Figure S2). mRNA coding for mPGES-2 was slightly but significantly increased in LDv as compared to healthy SV. The cyclooxygenases were also analysed at the protein levels (Figure S3), COX-2 was not detectable while a significant increase was shown for COX-1 content.

### Increased Content of the PGE_2_ Degrading Enzyme (15-PGDH) in Varicose Veins

Using Western blot analysis, a 29 kDa band corresponding to 15-PGDH was observed in the protein extracts of saphenous veins corresponding to the standard band (Ready control; Cayman). A significant increase (∼188%) was found in varicose veins (SDv and LDv; n = 5) as compared to healthy SV (n = 5; Figure 1C).

### Decreased PGE_2_ Content in Varicose Veins

EIA measurements showed significantly decreased PGE_2_ concentrations in the solution incubated for 24 h (Figure 1D) and for 30 min (Figure S4) with varicose veins (SDv and LDv; n = 5) as compared to healthy SV (n = 5). Interestingly, a progressive and significant decrease of the PGE_2_ levels was observed between healthy SV, SDv and LDv respectively. Similar results were found after 30 min incubation with arachidonic acid (1 µmol/L), in the presence or absence of glutathione (2.5 mmol/L) (data not shown, n = 3–4). This decreased content in PGE_2_ in varicose veins was associated with an increased production of TxA_2_ and PGD_2_, measured as their stable metabolites TxB_2_ and 15d-PGJ_2_, respectively (Figure S5).

### Decreased EP4 Receptor Content in Varicose Veins

**Figure 2:**
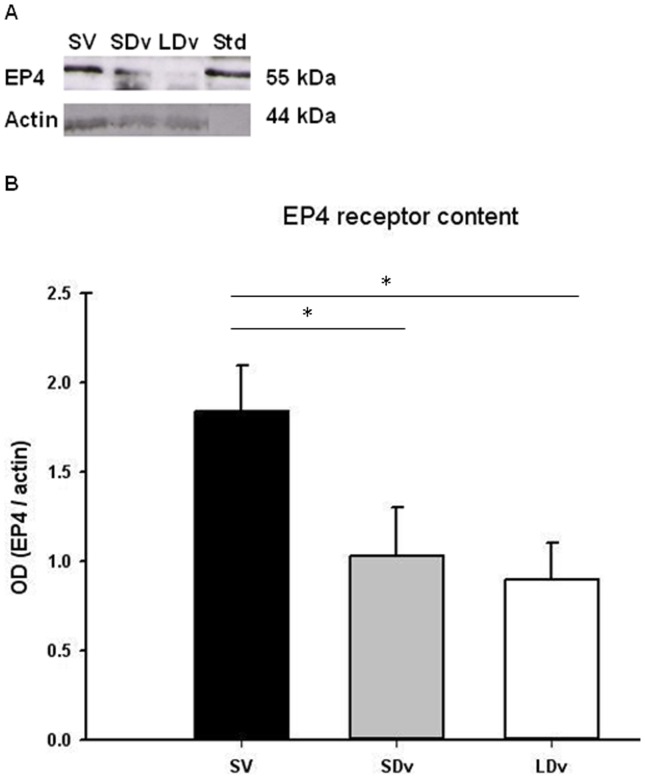
EP4 receptor decreased in varicose veins. Protein measurements, representative samples of Western blot (A) of EP4 receptor in human small and large diameter varicosities (paired SDv and LDv, n = 7) and healthy saphenous veins (SV, n = 4). Western blot quantification of EP4 (B). Optical density (OD, arbitrary units) was quantified by Scion Image® and normalized by α-actin; * P<0.05 as determined by one-way-ANOVA followed by the Tukey post-hoc test and by a paired t-test for varicose veins.

Using Western blot analysis, a strong band (about 55 kDa) was observed for the EP4 receptor (Figures 2 A, B) in the homogenates of saphenous veins. Protein density in varicose veins (SDv and LDv; n = 7) was significantly lower than in healthy SV (n = 4).

### Expression of MMPs and TIMPs

**Figure 3:**
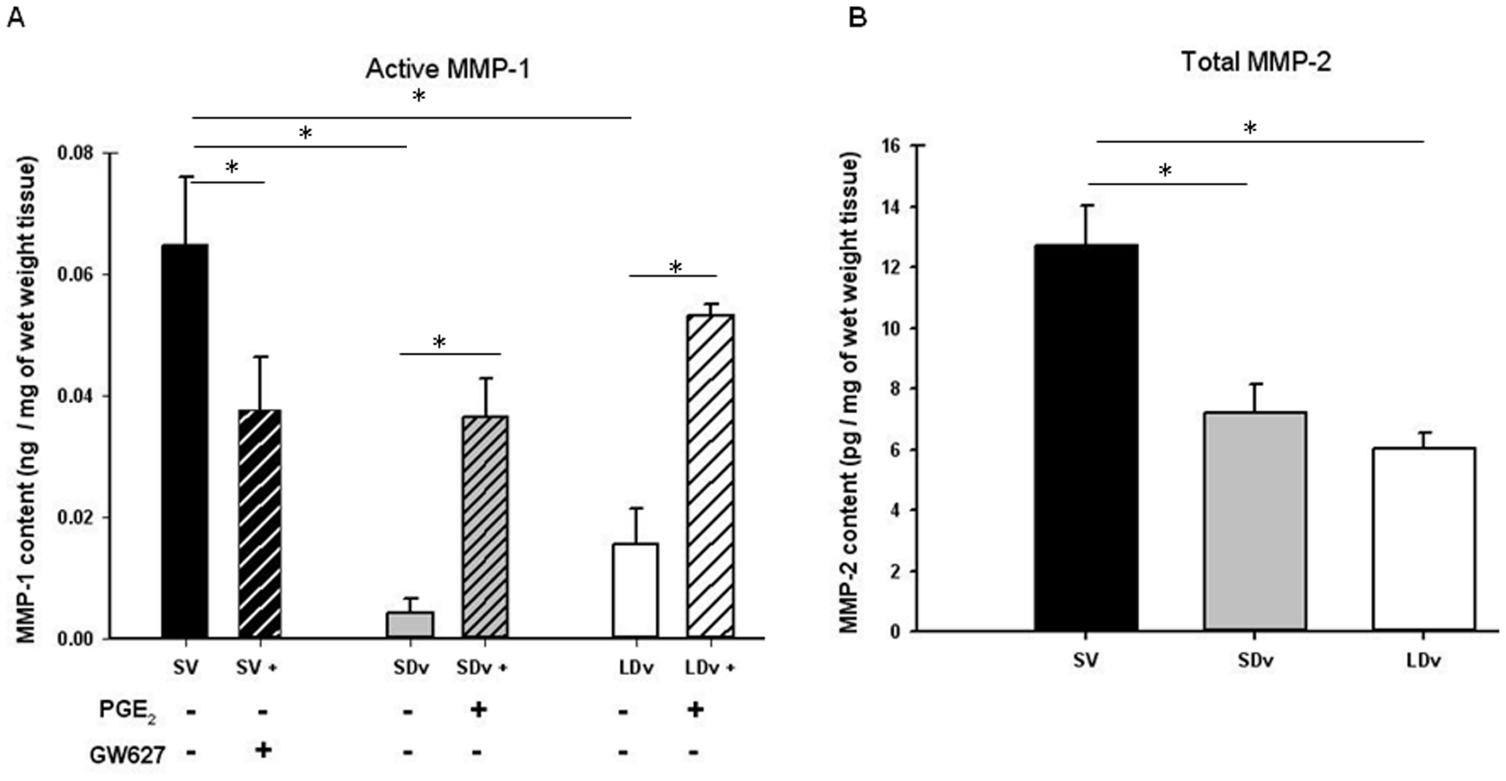
PGE_2_ is responsible for decreased MMP-1 activity. (A) Active MMP-1 content in human small and large diameter varicosities (paired SDv and LDv, n = 6) and healthy saphenous veins (SV, n = 4) after 24 h of incubation. Treatments with either PGE_2_ 10 µmol/L or GW62768X 1 µmol/L are represented by hatched bars. (B) Total MMP-2 content in varicose veins (n = 5) and SV (n = 4) after 30 min of incubation. Values were determined by EIA in supernatants and normalized by tissue wet weight; * P<0.05 as determined by one-way-ANOVA followed by the Tukey post-hoc test and by a paired t-test for varicose veins.

**Figure 4:**
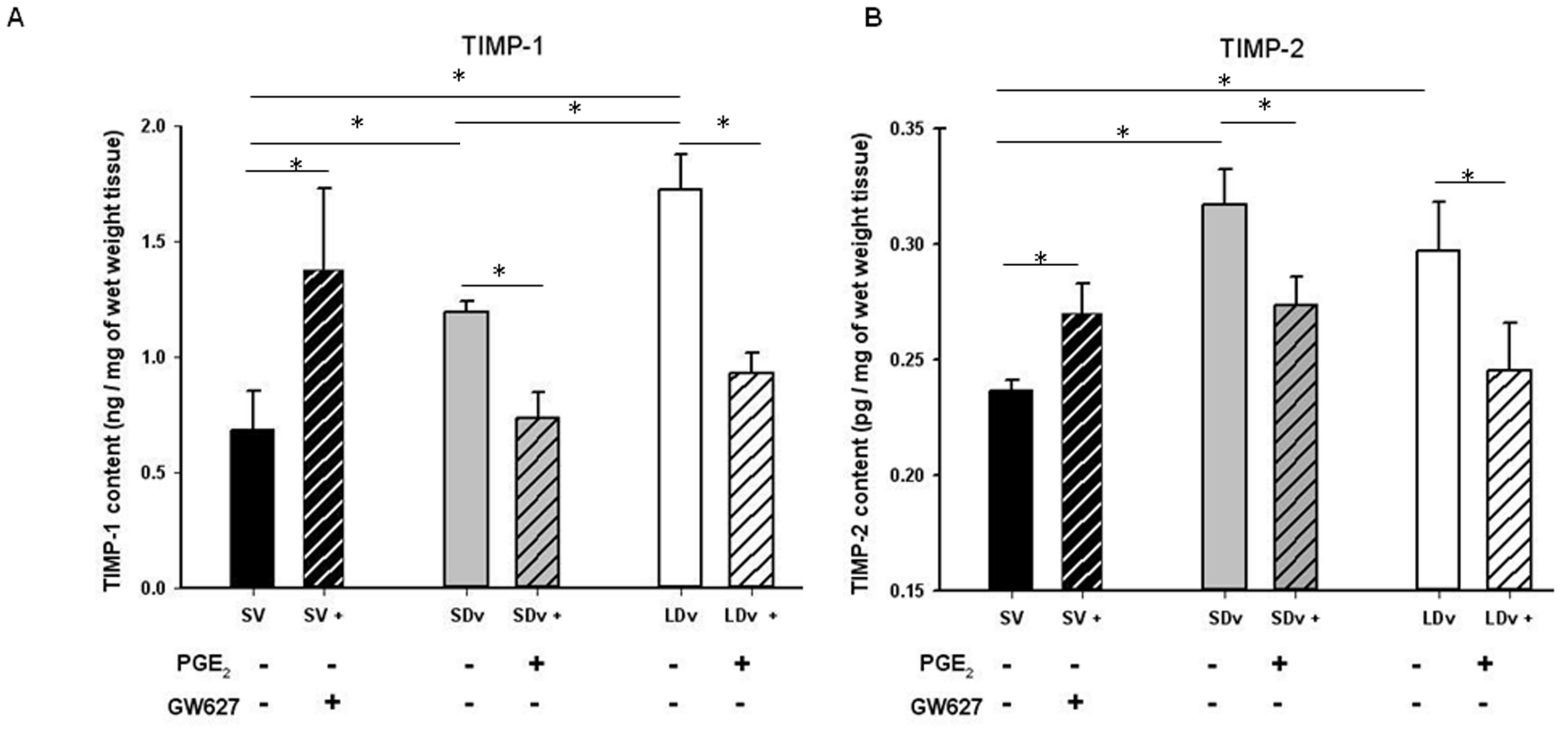
PGE_2_ is responsible for increased TIMP production. TIMP-1 (A) and TIMP-2 (B) contents in human small and large diameter varicosities (paired SDv and LDv, n = 6) and healthy saphenous veins (SV, n = 4). Treatments with either PGE_2_ 10 µmol/L or GW62768X 1 µmol/L are represented by hatched bars. Values were determined by EIA in supernatants after 24 h of incubation. Values are normalized by tissue wet weight; * P<0.05 as determined by one-way-ANOVA followed by the Tukey post-hoc test and by a paired t-test for varicose veins.

EIA measurements for MMP-1 and MMP-2 (Figures 3 A, B), TIMP-1 and TIMP-2 (Figures 4 A, B) were performed in the same samples to allow valid comparison and show significant changes. For MMP-1, measurements of pro-MMP and total MMP were made and the quantity of active MMP-1 was calculated. The subtraction showed a decrease in active MMP-1 in varicose veins (SDv and LDv; n = 6) as compared with healthy SV (n = 4). Incubation with PGE_2_ led to the activation of MMP-1 in varicose veins (SDv and LDv) to the same level as in healthy SV. The saphenous veins incubated with an EP4 antagonist showed a significant decrease in MMP-1 activation (Figure 3A). Total MMP-2 concentration was also measured in varicose veins (n = 5) and compared with healthy SV (n = 5) after a 30 min incubation. A significant decrease in MMP-2 expression was found in varicose veins (SDv and LDv) compared to healthy SV (Figure 3B). TIMP-1 measurement showed a significant increase in the varicose veins (SDv and LDv) compared to healthy SV (Figure 4A). Addition of PGE_2_ induced a decreased TIMP expression in varicose veins (SDv and LDv) whereas the healthy veins incubated with the EP4 antagonist showed a significant increase in TIMP. The EIA measurement for TIMP-2 (Figure 4B) showed similar results as for TIMP-1, a significant increase in its content in varicose veins, both small and large diameter, as compared to the healthy SV. For TIMP-1, we also observed a significant increase in its content in LDv as compared with SDv. Incubation with PGE_2_ significantly decreased TIMP-2 content in SDv and in LDv. In the case of healthy SV, incubation with the EP4 antagonist resulted in increased TIMP-2 content.

### Increased Collagen Content in Varicose Veins

**Figure 5:**
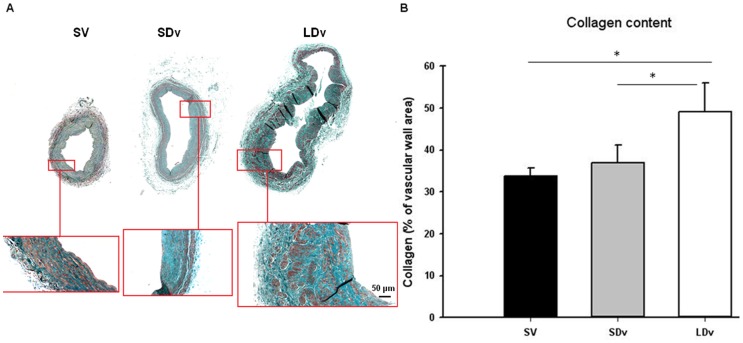
Increase of collagen content in varicose veins. Histomorphometric measurement of collagen content after Masson’s Trichrome staining (A) in human small and large diameter varicosities (paired SDv and LDv, n = 5) and healthy saphenous veins (magnificence 4X and 10X) (SV, n = 3). (B) Values obtained after Photoshop measurement of colour density. Values are normalized by total surface area; * P<0.05 as determined by one-way-ANOVA followed by the Tukey post-hoc test and by a paired t-test for varicose veins.

Using histomorphometry, collagen content was measured on cross sections of healthy (n = 3) and varicose (n = 5) SV (Figures 5 A, B). A significantly higher content of collagen was found in LDv as compared to healthy SV and SDv.

## Discussion

Our results demonstrate that in the varicose veins, PGE_2_ plays a major role in vascular wall remodeling and in collagen over-expression. The reduced synthesis (*via* decreased mPGES-1) and/or increased degradation (*via* increased 15-PGDH) of PGE_2_ are responsible for its lower concentration in human varicose saphenous veins when compared to healthy ones. This reduced PGE_2_ content and the lower density of its receptor (EP4) are responsible for the down-regulation of the MMP/TIMP ratio in varicose veins. The consequence of this biological cascade is a reduction of active collagenase content and an accumulation of collagen in the vascular wall of varicose veins and could explain the intima hyperplasia and the thickening observed in the varicose wall [4], [37]. This phenomenon is in complete accordance with a recent publication where selective deletion of mPGES-1 in both endothelial and vascular smooth muscle cells resulted in hyperplasia by enhancing the neointimal proliferative response to vascular injury in mice [38].

In human saphenous vein preparations, PGES are coupled only with the constitutive COX-1 since COX-2 was not detectable [19] and this kind of coupling was also previously observed in non-inflammatory conditions. [15] In our study, protein (Figure 1) and mRNA (Figure S2) levels of the different PGES isoforms were analysed. cPGES protein was not found in all sample and was only detectable at the mRNA level. In contrast, mPGES-2 protein was found at similar levels in all venous preparations. Our results concerning mPGES-1 were unexpected since we found this enzyme in the SV while mPGES-1 expression is classically induced by inflammatory conditions *via* NF-κB. [9] However, this isoform has also been found as a constitutive enzyme in some human cell types, [18], [39] such as fibroblasts, [40] the astroglioma cell line [41] or vascular smooth muscle cells. [12] In these studies, positive correlations between the presence of mPGES-1 and a higher level of PGE_2_ are shown. We also showed a significant decrease (50–75%) in mPGES-1, at both protein and mRNA levels, in LDv compared to the other segments (SV and SDv). This decreased content could be due to several mechanisms such as an increased of PGD_2_ metabolite, 15d-PGJ_2_ (as observed in Figure S5) which is known to down regulate mPGES-1 [42] or by other endogenous regulator. In addition, we found that 15-PGDH content was increased in varicose veins (SDv and LDv). Taken together, our measurements of these enzymes involved in the synthesis and degradation of PGE_2_ are in favour of a reduced concentration of this prostaglandin in varicose veins. This effect is not due to a decreased expression of COX-1 since the protein content was increased (Supplementary data). This dysregulation of PGE_2_ production led to a reorganisation of PG synthesis such as an increased thromboxane A_2_ and PGD_2_ production (Figure S5).

In our human saphenous vein preparations, the accumulation of released PGE_2_ is in the “pg/mg of tissue” range after 30 min incubation as previously described [43] and greater (1000 fold) after 24 h incubation as previously shown. [44] However, in both cases, in comparison to SV, similar and significant reductions in PGE_2_ concentrations were found, i.e. 22–23% and 44–59% in the supernatant of SDv and LDv, respectively. These decreases in PGE_2_ concentration in SDv, and even more so in LDv, correlate with the severity of the pathology and are in complete accordance with our results concerning the expression of the enzymes responsible for PGE_2_ metabolism. More precisely, in SDv, only increased 15-PGDH expression could explain the decrease in PGE_2,_ while in LDv, both the increase in15-PGDH and the decrease in mPGES-1 may participate in the more pronounced decrease in PGE_2_ concentration. An inverse regulation has been observed in human pancreatic tumors where enhanced PGE_2_ production proceeds via the over-expressions of COX-2 and microsomal PGES-1 and the down-regulation of 15-PGDH by SNAI2. [45] In addition, the lower concentrations of PGE_2_ measured in varicose veins will have lower EP4-mediated effects since a significant decrease in this receptor density was found in varicose (SDv and LDv) as compared with SV. For example, the EP4-mediated vasodilation previously reported in human healthy saphenous veins [43] should be reduced in varicose veins. Similarly, MMP expression could be modified by decreases in both PGE_2_ and EP4 receptor expression.

MMP -1, -2, -9, some enzymes responsible for ECM degradation, and/or their inhibitors TIMP-1, -2, have been measured in human varicose veins and the results of many studies are controversial (see reviews [2], [34]). MMP quantity or activity was shown to be increased or decreased in varicose veins as compared to healthy SV. In these publications, the varicose vein preparations originated from different locations and from patients with various clinical stages of chronic venous disease ranging from C1 to C6, facts which could explain the variable results described. In many of the studies, only total MMP was quantified, there was no distinction between the pro- and active- MMP and the calculations of the ratio active-MMP versus TIMP were even rarer. Our results describe a decrease in active MMP-1, total MMP-2 (while total MMP-1 is not modified in varicose veins) and an increase in TIMP-1 and TIMP-2 concentrations in varicose veins. They are in accordance with previously reported measurements in the same tissues [33], [46], [47] and several immunohistochemistry experiments. [48], [49], [50].

The relation between PGE_2_ and MMPs has been demonstrated in some vessel types but there is no report in human saphenous veins. Our experiments are in favour of a clear relation between PGE_2_ and MMP activation, a process that is decreased in human varicose veins. In healthy SV, the addition of the EP4 antagonist, GW627368X, produced both a decrease in active MMP-1 and an increase in TIMP-1/-2 concentrations; a reverse effect was observed in varicose veins with exogenous PGE_2_ stimulation. These MMP/TIMP expressions regulated by PGE_2_ are mostly dependent upon EP4 receptor activation, as suggested by the GW627368X antagonism. However, PGE_2_ or EP4 antagonist did not totally restore or abolish the activation of MMP-1, respectively. It is unlikely that the EP2 receptor could be involved since, EP2 mRNA, was not found in human saphenous vein. [43] Other pathways, such as Reactive Oxygen Species**,** could be implicated. [51].

Our results on the histological quantification of collagen, a major vascular wall component, support our results on MMP/TIMP regulation between human healthy and varicose veins. Masson’s trichrome staining showed significant increases in collagen content in the LDv where MMP-1, the enzyme responsible for its degradation, and to a lesser extent, MMP-2, are significantly reduced as compared to healthy SV. Furthermore, this result is in accordance with previous studies where investigators found an increase in type I collagen content in segments of varicose veins compared to normal veins. Hydroxyproline, the major amino acid of collagen was also augmented. [30], [52], [53].

In contrast to varicose veins, in many other human vascular pathologies the reverse is observed: vascular wall remodeling is associated with an increased PGE_2_ synthesis. Gomez-Hernandez *et al*. [26] demonstrated that in the plasma of patients with acute coronary syndrome, higher plasma PGE_2_ concentrations were found and these correlated with MMP-9 activity. In human atherosclerotic plaques, mPGES-1 and EP4 receptors are over-expressed. [27], [54], [55] Similarly, studies on the vascular wall of human aneurysms showed an increase in mPGES-1 expression, [56] in PGE_2_ production [57] and in EP4-receptor presence. [22] A selective EP4-receptor agonist (ONO-329-AE1) increased the activation of MMP-2 in human aortic aneurysm explants. [22] Finally, changes in the vascular wall composition and formation of aortic aneurysms could be prevented in mice knocked-out for mPGES-1 or EP4-receptors. [22], [58], [59] This discrepancy between these other vascular pathologies and human varicose vein pathology could be explained by the inflammatory environment which is absent only in the latter case. [19] Although in the above-mentioned vascular pathologies and in varicose veins an opposite process is displayed, overall, these studies showed a control of vascular wall remodeling *via* PGE_2_ metabolism and EP4 receptor stimulation.

In conclusion, the reduction of PGE_2_ concentrations in human varicose veins is due to a decrease in mPGES-1 and an increase in 15-PGDH. These effects lead to the imbalance of vascular wall remodeling by decreasing the MMP/TIMP ratio (Table 1) and could result in the accumulation of collagen in varicose veins. This endogenous mechanism could be a protective effect of the saphenous vein in order to restrain the blood stasis by reinforcing the vascular wall, avoiding ectasic segment formation and venous wall rupture.

**Table 1:** Ratio (Active MMP-1)/TIMP-1 and TIMP-2.

Tissue	(active MMP-1)/TIMP-1	(active MMP-1)/TIMP-2
**SV n = 4**	0.10±0.024	254.13±75.92
**SDv n = 6**	0.008±0.003*	40.19±14.48*
**LDv n = 6**	0.011±0.002*	59.43±10.55*

Measurements were obtained by ELISA for all human small and large diameter varicosities (paired SDv and LDv) and healthy saphenous veins (SV). Values are obtained after division of active MMP-1 values by TIMP-1 and TIMP-2 values normalized per mg of weight tissue. Statistical analysis was performed using one-way-ANOVA followed by the Tukey post-hoc test or by paired t test.

*indicates a significant difference (P<0.05) with SV.

## Supporting Information

Figure S1 **cPGES expression in human internal mammary artery (IMA**). (A) Histogram represent western blot analysis for internal mammary arteries (IMA, n = 3) for cPGES. (B) Representative samples. Optical density (OD, arbitrary units) was measured by Scion Image® and the mean normalized by actin.
(TIF)

Figure S2 **Dysregulation in PGES mRNA.** mRNA expression in human small and large diameter varicosities (paired SDv and LDv, n = 5) and healthy saphenous veins (SV, n = 5). mRNA levels of prostaglandin E synthases (mPGES-1, mPGES-2 or cPGES) were determined by Real-Time PCR and normalized by glyceraldehyde-3-phosphate dehydrogenase (GAPDH) mRNA level; * P<0.05 as determined by one-way-ANOVA followed by the Tukey post-hoc test and by a paired t-test for varicose veins.
(TIF)

Figure S3 **Increased COX-1 protein in varicose veins.** Protein measurements (A), representative samples of Western blot of cyclooxygenases (COX-1 or COX-2) in human small and large diameter varicosities (paired SDv and LDv, n = 6) and healthy saphenous veins (SV, n = 4). Standards (Std) are Western ready controls from Cayman. Histogram (B) represents Western blot quantification of COX-1 corresponding band. Optical density (OD, arbitrary units) was measured by Scion Image® and the mean normalized by actin; * P<0.05 as determined by one-way-ANOVA followed by the Tukey post-hoc test and by a paired t-test for varicose veins.
(TIF)

Figure S4 **Decreased PGE2 content in 30 min.** PGE_2_ content in human small and large diameter varicosities (paired SDv and LDv, n = 8) and healthy saphenous veins (SV, n = 4). Values were determined by EIA in supernatants after 30 min of incubation of the venous preparations in Tyrode solution. Results are normalized by tissue wet weight; * P<0.05 as determined by one-way-ANOVA followed by the Tukey post-hoc test and by a paired t-test for varicose veins.
(TIF)

Figure S5 **Prostanoids expression.** Thromboxane (Tx) B_2_ and 15d-PGJ_2_ content in human small and large diameter varicosities (paired SDv and LDv, n = 5) and healthy saphenous veins (SV, n = 5). Values were determined by EIA in supernatants after 24 hours of incubation of the venous preparations in RPMI solution. Results are normalized by tissue wet weight; * P<0.05 as determined by one-way-ANOVA followed by the Tukey post-hoc test and by a paired t-test for varicose veins.
(TIF)

File S1 **Supporting information about experimentals protocols.** Table S1. Primers used for Real-Time PCR, s: sense, as: anti sense.
(DOC)
