# Supplementary figures and images for: Decreased PGE2 Content Reduces MMP-1 Activity and Consequently Increases Collagen Density in Human Varicose Vein
Source: PLoS One. 2014 Feb 5;9(2):e88021. doi: 10.1371/journal.pone.0088021 (PMC3914898; doi:10.1371/journal.pone.0088021)

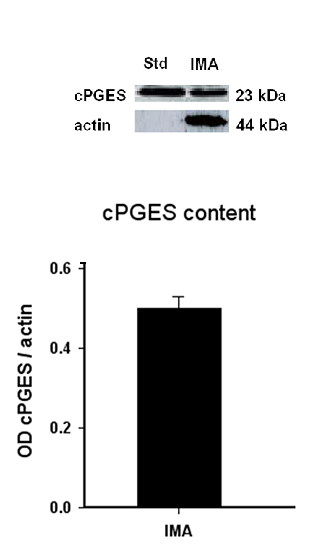

Supplement: Figure S1 — cPGES expression in human internal mammary artery (IMA). (A) Histogram represent western blot analysis for internal mammary arteries (IMA, n = 3) for cPGES. (B) Representative samples. Optical density (OD, arbitrary units) was measured by Scion Image® and the mean normalized by actin. (TIF) [file pone.0088021.s001.tif]

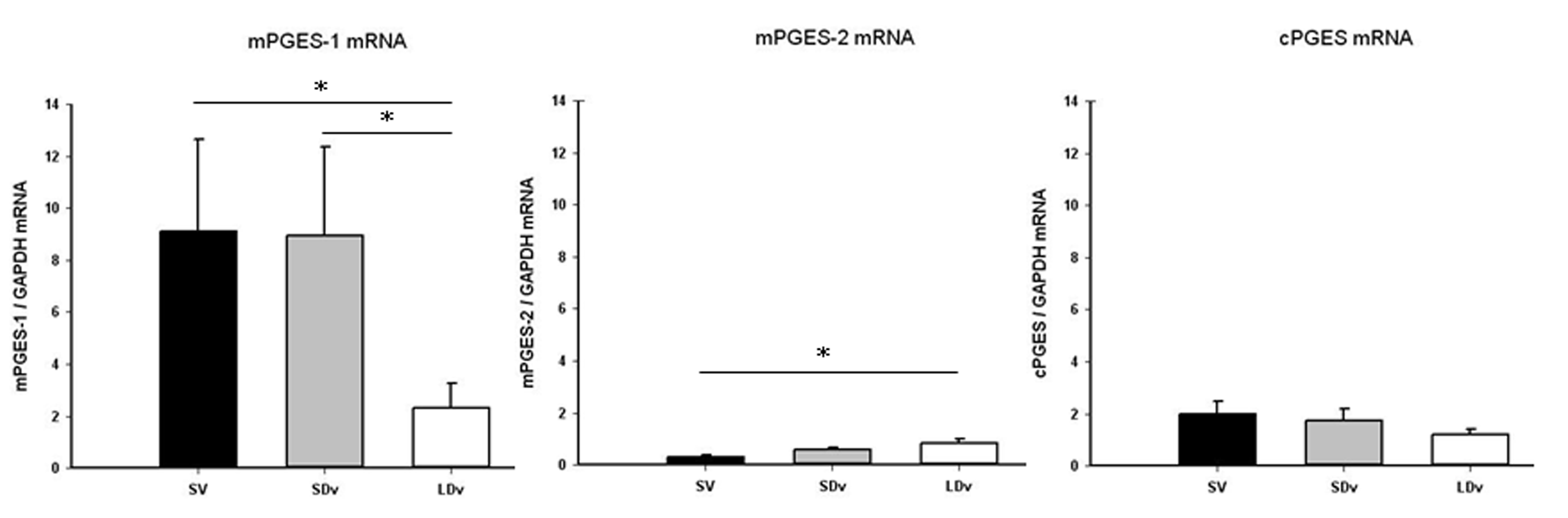

Supplement: Figure S2 — Dysregulation in PGES mRNA. mRNA expression in human small and large diameter varicosities (paired SDv and LDv, n = 5) and healthy saphenous veins (SV, n = 5). mRNA levels of prostaglandin E synthases (mPGES-1, mPGES-2 or cPGES) were determined by Real-Time PCR and normalized by glyceraldehyde-3-phosphate dehydrogenase (GAPDH) mRNA level; * P<0.05 as determined by one-way-ANOVA followed by the Tukey post-hoc test and by a paired t-test for varicose veins. (TIF) [file pone.0088021.s002.tif]

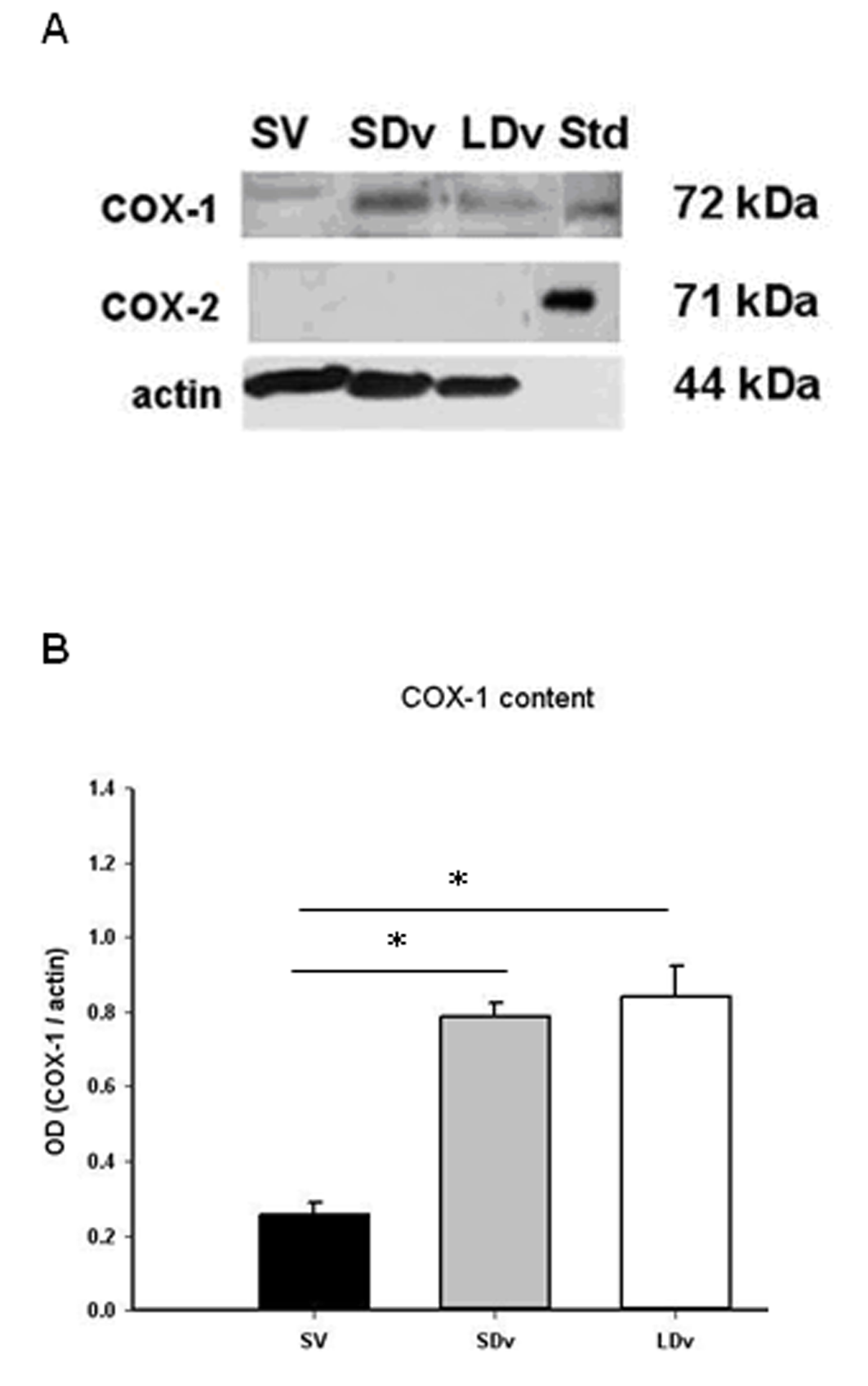

Supplement: Figure S3 — Increased COX-1 protein in varicose veins. Protein measurements (A), representative samples of Western blot of cyclooxygenases (COX-1 or COX-2) in human small and large diameter varicosities (paired SDv and LDv, n = 6) and healthy saphenous veins (SV, n = 4). Standards (Std) are Western ready controls from Cayman. Histogram (B) represents Western blot quantification of COX-1 corresponding band. Optical density (OD, arbitrary units) was measured by Scion Image® and the mean normalized by actin; * P<0.05 as determined by one-way-ANOVA followed by the Tukey post-hoc test and by a paired t-test for varicose veins. (TIF) [file pone.0088021.s003.tif]

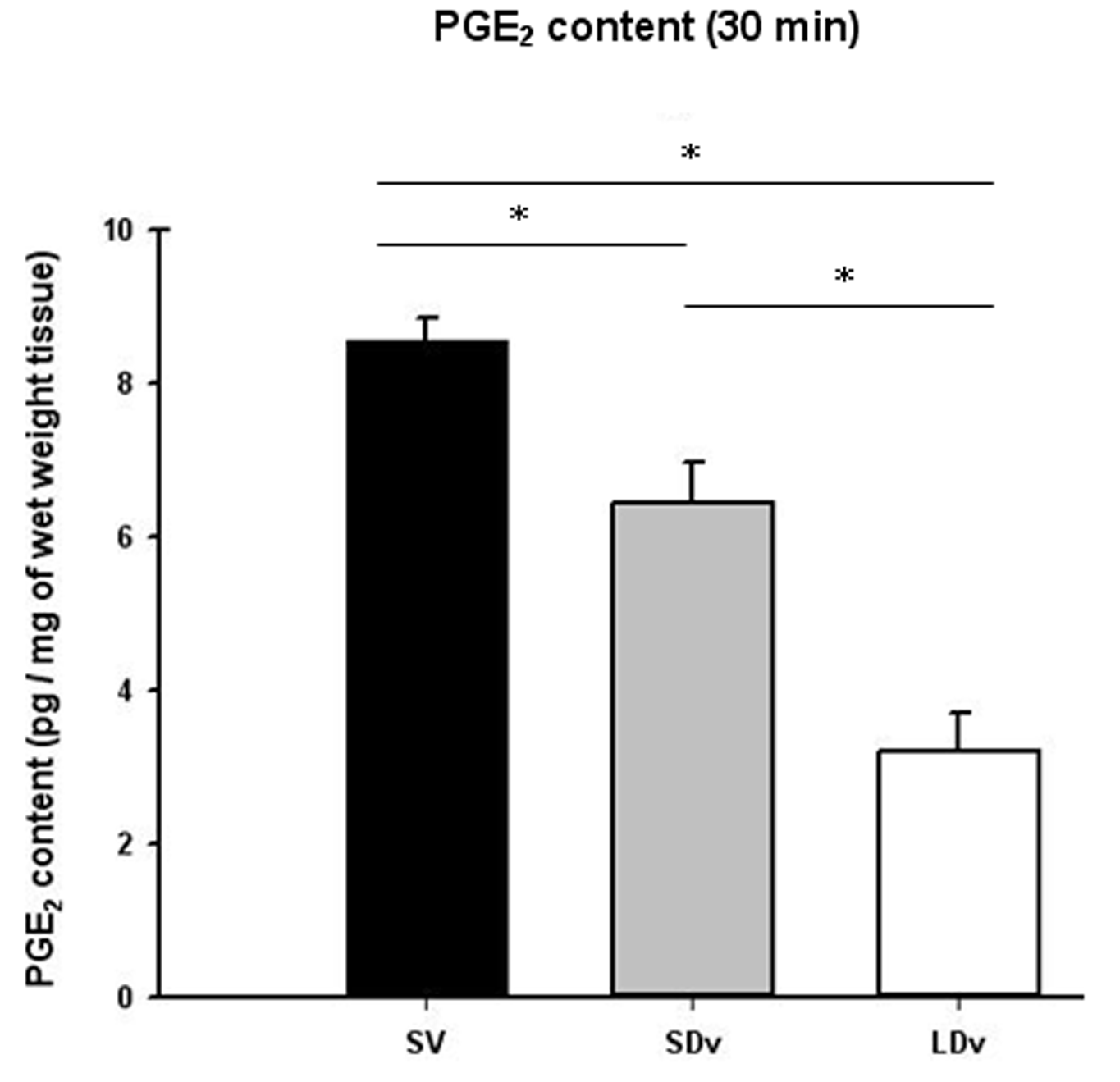

Supplement: Figure S4 — Decreased PGE2 content in 30 min. PGE2 content in human small and large diameter varicosities (paired SDv and LDv, n = 8) and healthy saphenous veins (SV, n = 4). Values were determined by EIA in supernatants after 30 min of incubation of the venous preparations in Tyrode solution. Results are normalized by tissue wet weight; * P<0.05 as determined by one-way-ANOVA followed by the Tukey post-hoc test and by a paired t-test for varicose veins. (TIF) [file pone.0088021.s004.tif]

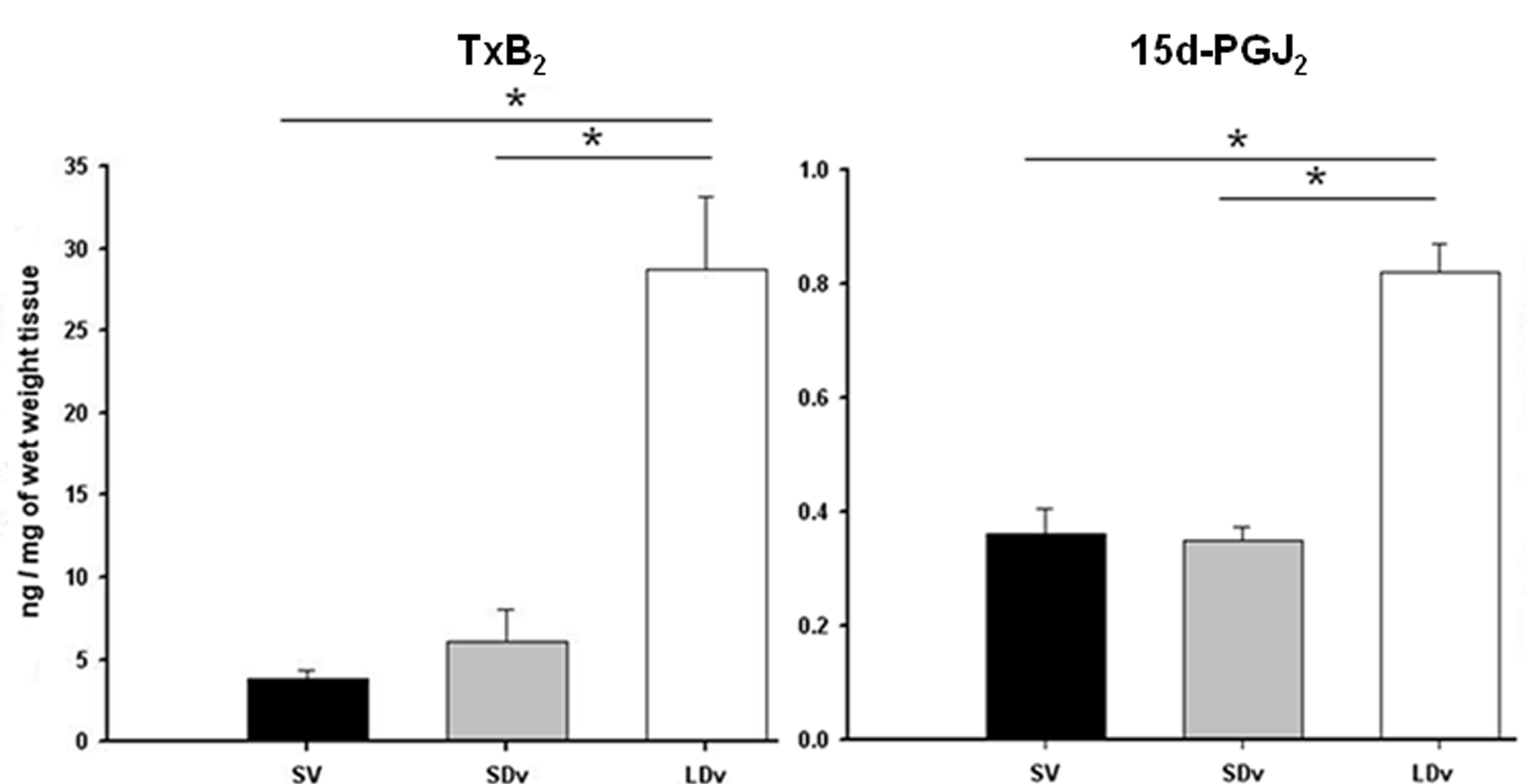

Supplement: Figure S5 — Prostanoids expression. Thromboxane (Tx) B2 and 15d-PGJ2 content in human small and large diameter varicosities (paired SDv and LDv, n = 5) and healthy saphenous veins (SV, n = 5). Values were determined by EIA in supernatants after 24 hours of incubation of the venous preparations in RPMI solution. Results are normalized by tissue wet weight; * P<0.05 as determined by one-way-ANOVA followed by the Tukey post-hoc test and by a paired t-test for varicose veins. (TIF) [file pone.0088021.s005.tif]
