# Supplementary material for: Decreased PGE2 Content Reduces MMP-1 Activity and Consequently Increases Collagen Density in Human Varicose Vein
Source: PLoS One. 2014 Feb 5;9(2):e88021. doi: 10.1371/journal.pone.0088021 (PMC3914898; doi:10.1371/journal.pone.0088021)
Supplement: File S1 — Supporting information about experimentals protocols. Table S1. Primers used for Real-Time PCR, s: sense, as: anti sense. (DOC) [file pone.0088021.s006.doc]

**Supporting Information**

**7 Western blot analysis**

Specific antibodies used: mPGES-1 (monoclonal antibody (mAb)) (Oxford Bio Therapeutics, Oxford, UK)[^35^](#_ENREF_35); cPGES (mAb), COX-1 (mAb), COX-2 (mAb), mPGES-2 (polyclonal antibody (pAb)), EP4 (pAb), 15-PGDH (pAb) (Cayman), cPGES (pAb; Thermo Scientific, Rockford, IL USA), COX-1 (mAb; Santa-Cruz Biotechnology, Santa Cruz, CA, USA) and α actin (mAb) (Dako, Glostrup, Denmark), diluted respectively at 1:1, 000, 1:250, 1:200, 1:1, 000, 1:250, 1:500, 1:500, 1:1000, 1:500 and 1:5000 in TBS. Subsequently, the membranes were incubated with the appropriate horseradish peroxidase-labelled secondary antibody (anti-Mouse IgG from Sigma-Aldrich or anti-Rabbit IgG from Jackson Immuno Research, West Grove, PA, USA).

**8 mRNA expression measurement**

**8.1 Reverse-transcription**

Total RNAs were extracted using the Tissue RNA Kit (Omega Bio-tek, Norcross, GA, USA), according to the manufacturer’s instructions. Reverse transcription was conducted for 90 min at 37°C using 0.2 μg RNA in 20 μl of reaction mixture, 200 U M-MLV reverse transcriptase, and Oligo-d(T)12-18 (Invitrogen, Grand Island, NY, USA).

**8.2 Real-Time PCR analysis of mPGES-1, mPGES-2 and cPGES mRNA**

Expression levels of mPGES-1, mPGES-2 and cPGES mRNA in varicose veins and healthy SV were determined by quantitative Real-Time PCR (Bio-rad, Hercules, CA, USA, CFX96) analysis. The PCR primer sequences for targeted genes are summarized in Table 1**.** A glyceraldehyde 3-phosphate dehydrogenase (GAPDH) primer/probe set was used in separate wells as internal control for input cDNA. Thermal cycling conditions included 3 min at 95°C, followed by 40 cycles for 15 s at 59°C and 15 s at 72°C. Real-time fluorescence measurements and cycle threshold values were determined. Expression levels of each PGES isomer and GAPDH mRNA were calculated using Primer Express software. To control total cDNA input variations, mPGES-1, mPGES-2 and cPGES mRNA levels for each sample were normalized to GAPDH mRNA levels and expressed as arbitrary units.

**9 PGE_2_ content**

Before enzyme immunoassay (EIA) measurement of PGE_2_, fresh vein samples were incubated for 30 min in Tyrode’s solution [(concentrations in mmol/L): 139.2 NaCl; 2.7 KCl: 1.8 CaCl_2_; 0.49 MgCl_2_; 11.9 NaHCO_3_: 0.4 NaH_2_PO_4_; 5.5 glucose, gassed with CO_2_ 5% and O_2_ 95%, pH: 7.4] (70 mg wet tissue / mL solution) with or without treatment. Supernatants and tissue were harvested and frozen until use.

**10 Prostanoids content**

The concentration of TxB_2_ and 15d-PGJ_2_ in fresh dissected healthy SV or varicose veins was determined using an enzyme immunoassay (EIA) kit (Cayman). Veins were incubated for 24h in RPMI solution with antibiotic at 37°C with 5% CO_2_. The supernatants were obtained and diluted with the assay buffer. The prostanoids concentrations were determined according to the instructions provided with the kit.

**11 Normalization**

In order to confirm that α-actin protein does not change in the pathological situation, α-actin content was checked by correlating the total protein content determined by BCA assay before Western blot with α-actin optical density (OD) obtained after Western blot by Scion Image®. A significant correlation was found and no significant difference was observed between the three types of saphenous vein used (n=7-9). In view of this result, we normalized either by α-actin or protein content.

**Table S1:** PCR Primers

| **Gene** | **Sequences** |
| --- | --- |
| mPGES1 s | 5’ cttcctcgtgggccgtgt 3’ |
| mPGES1 as | 3’ tgggccagggtgtaggtc 5’ |
| mPGES2 s | 5’ agcgccctcaagacctacctggtgt 3’ |
| mPGES2 as | 3’ ccactgccgccacttcatctcct 5’ |
| cPGES s | 5’ aagactgggaagatgattcagatg 3’ |
| cPGES as | 3’ cctcatcaccacccatgttgttc 5’ |
| GAPDH s | 5’ gggcaccctgggctaaactga 3’ |
| GAPDH as | 3’ tgctcttgctggggctggt 3’ |
